# Exploiting plant immune “switches” for resistance engineering

**DOI:** 10.1007/s44154-026-00306-4

**Published:** 2026-04-24

**Authors:** Bangting Wu, Kaichen Xu, Anum Bashir, Xinyu Han, Qiping Sun, Peng Sun, Guan-Feng Wang, Ricky J. Milne, Meixiang Zhang, Leiyun Yang, Guoyong Xu, Guotian Li

**Affiliations:** 1 National Key Laboratory of Agricultural Microbiology, Hubei Hongshan Laboratory, Hubei Key Laboratory of Plant Pathology, The Center of Crop Nanobiotechnology, Huazhong Agricultural University, Wuhan, 430070 China; 2 State Key Laboratory for Biology of Plant Diseases and Insect Pests-Key Laboratory of Control of 10 Biological Hazard Factors (Plant Origin) for Agri-Product Quality and Safety, Ministry of Agriculture, Institute of Plant Protection, Chinese Academy of Agricultural Sciences, Beijing, China; 3 The Key Laboratory of Plant Development and Environmental Adaptation Biology, Ministry of Education, School of Life Sciences, Shandong University, Qingdao, 266237 Shandong China; 4 CSIRO Agriculture and Food, Canberra, ACT 2601 Australia; 5 College of Life Sciences, Shaanxi Normal University, Xian, 710119 China; 6 State Key Laboratory of Agricultural and Forestry Biosecurity, College of Plant Protection, Nanjing Agricultural University, Nanjing, 210095 China; 7 State Key Laboratory of Hybrid Rice, Institute for Advanced Studies (IAS), Hubei Hongshan Laboratory, RNA Institute, Wuhan University, Wuhan, 430072 China

**Keywords:** Immune “switches”, Resistance engineering, Engineering promoters, Engineering mRNA, Engineering proteins

## Abstract

Plant diseases, caused by various pathogens, pose a serious threat to sustainable agriculture. Plants have evolved a sophisticated immune system to detect and mount effective responses against pathogens. The plant immune system contains numerous programmable immune "switches" that safeguard plants against pathogens while minimizing energy consumption in the absence of pathogens. The cloning of disease-resistance (*R*) genes, along with the elucidation of the molecular mechanisms regulating these immune "switches", has provided resources and strategies for genetic engineering of novel disease resistance. This review focuses on the resistance engineering through the manipulation of immune “switches”. It begins by summarizing recent advances in plant immunity, then explores the mechanisms behind plant immune "switches", and finally discusses potential strategies for engineering immune genes via these "switches". These strategies include engineering promoters to confer precise spatiotemporal regulation at the transcriptional level; engineering mRNA or its regulatory elements to facilitate specific and stable translation of R proteins at the post-transcriptional and translational levels; and engineering proteins at the post-translational level to broaden the pathogen-recognition spectrum of R proteins. Collectively, these strategies will accelerate the development of disease-resistant crop cultivars, thereby enhancing agricultural productivity and global food security.

## Introduction

Plant diseases, caused by a wide range of pathogens (including viruses, bacteria, fungi, oomycetes, and nematodes), account for 10–20% of annual global crop losses. This in turn results in economic damages exceeding $220 billion each year and poses a severe threat to global food security (Hossain et al. 2024). Resistance genes are critical for the environmentally friendly control of crop diseases. In addition to the well-known disease resistance, which prevents or eradicates the pathogen, disease tolerance is another successful and sufficient immune strategy that allows the pathogen to persist while mitigating the associated tissue damages, thereby safeguarding crop growth and yield (Tang et al. 2025a, b; Tang et al. 2024). The immune strategy of disease resistance operates through two interconnected layers that function as an integrated defense network rather than separate pathways. Pattern-triggered immunity (PTI) is initiated upon the recognition of pathogen-associated molecular patterns (PAMPs) by pattern-recognition receptors (PRRs). Concurrently, PTI signaling leads to the biosynthesis of salicylic acid (SA), a key phytohormone in plant immunity. SA accumulation promotes the redox-dependent monomerization of NPR1, enabling its translocation into the nucleus. There, NPR1 interacts with TGA transcription factors to activate the expression of downstream defense genes, including *RBOHD*, *PR1* and *PR2* (Cao et al. 2024; Fu and Dong 2013; Han et al. 2025a, b). Effector-triggered immunity (ETI), triggered by nucleotide-binding leucine-rich repeat (LRR) receptors (NLRs) that detect specific pathogen effectors, mounts more robust immune responses. ETI enhances PTI responses by increasing transcript levels of *PRRs*, *RBOHD*, and *BIK1*, while PTI provides the foundation for ETI activation through shared RLCKs, RBOHD phosphorylation and calcium signaling (Huang et al. 2023; Kimura et al. 2012). ETI potentiates the ROS burst via RBOHD, facilitates the assembly of EDS1/PAD4/ADR1 helper complexes, and amplifies SA-dependent transcriptional reprogramming, ultimately triggering localized cell death and systemic acquired resistance (Huang et al. 2023; Kong et al. 2016). Thus, PTI and ETI constitute a continuum of immune responses that collectively provide broad-spectrum basal defense and pathogen-specific immunity (Ngou et al. 2021; Yuan et al. 2021). In agriculture, the application of disease-resistance (*R*) genes has significantly reduced losses caused by various diseases, thereby safeguarding food security.

Despite the sophistication of the plant immune systems, significant challenges remain for engineered resistance. The foremost among these is the growth-defense trade-off. For instance, while timely immune activation is crucial for survival, mistimed or constitutive immune responses can lead to autoimmunity and severe growth penalties. This conflict necessitates that the introduction of a new *R* gene into plants should be precisely regulated on the DNA, mRNA, and protein levels (Hou and Xu 2025), which is compounded by the vulnerability of immune recognition, as pathogens continuously evolve to evade or suppress plant defense. For example, the rice gene *Xa23* encodes an executor R protein that confers resistance to bacterial blight, caused by *Xanthomonas oryzae* pv*. oryzae* (*Xoo*). Its activation by the transcription activator-like effector (TALE) AvrXa23 triggers robust immune responses (Wang et al. 2014a, b). However, mutant variants of AvrXa23 have evolved to bypass recognition by *Xa23*, allowing the pathogen to overcome resistance (Xu et al. 2022). Additionally, genetic variation among different plant species limits the transferability of immune receptors across species, as recipient plants may lack essential “helper” receptors or compatible immune signaling components. These limitations highlight the urgent need for innovative strategies to engineer broad-spectrum disease resistance by repurposing immune “switches” to bypass these evolutionary and physiological constraints. Central to this effort is the understanding and manipulation of plant immune “switches”, including the ability to distinguish “friend from foe” and the regulatory nodes that toggle defense responses on and off to balance immunity and growth.

With advances in biotechnology and artificial intelligence (AI), plant immunity research is entering a new era. We can now not only “repurpose” plants' innate immune pathways but also leverage the core principles of gene circuit engineering for “programmable” regulation of plant immunity (Borowsky and Bailey-Serres 2024). AI-based CRISPR-Cas technologies enable targeted modifications in both coding and non-coding genomic regions. Mutations within coding sequences can alter protein conformation or enzymatic activity, thereby regulating endogenous signaling and metabolism while changes within non-coding regions, such as modifications to transcription factor binding sites, can fine-tune endogenous gene expression. These innovations signify a paradigm shift in crop protection, ushering in an era of rational and precise genetic engineering. Consequently, broad-spectrum disease-resistant crop varieties are anticipated to be engineered, aligning with global goals for sustainable agriculture. In this transformation, immune "switches" represent central regulatory nodes for disease resistance programming. In this review, we discuss these immune switches at the transcriptional, post-transcriptional, translational, and post-translational levels, offering strategies for engineering disease resistance in plants.

## Transcriptional level engineering

**Fig. 1:**
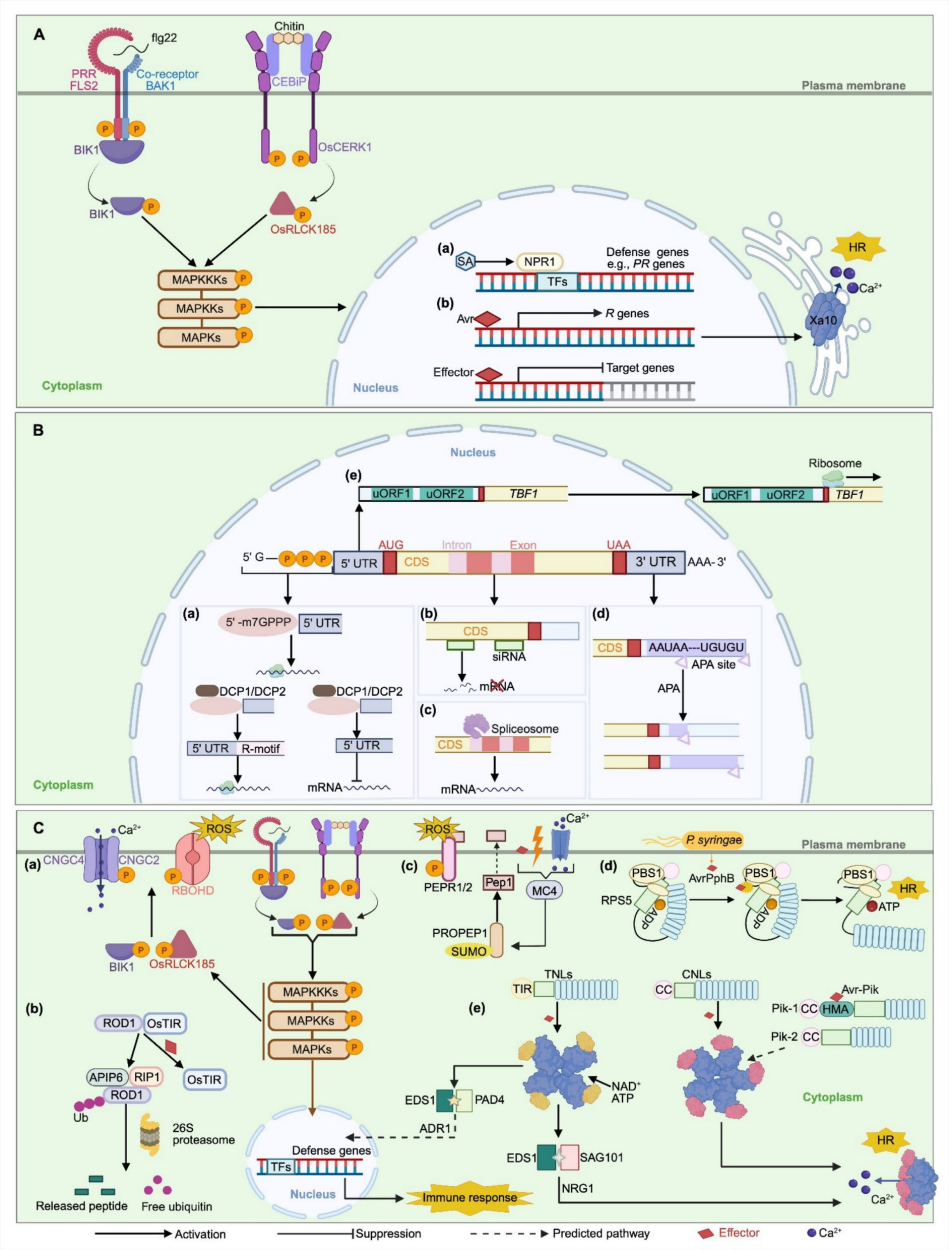
The regulatory networks of plant immunity. **A** Transcriptional regulation of plant immunity. **a.** In pattern-triggered immunity (PTI), recognition of pathogen-associated molecular patterns (PAMPs) by surface-localized pattern-recognition receptors (PRRs) activates mitogen-activated protein (MAPK) cascades, leading to phosphorylation and activation of WRKY transcription factors. These WRKYs stimulate salicylic acid (SA) biosynthesis, which, in turn, promotes NPR1 monomerization and nuclear translocation. Within the nucleus, NPR1 interacts with TGA transcription factors to activate SA-responsive defense genes such as *PR1* and *PR2*, reinforcing basal resistance. **b.** In effector-triggered immunity (ETI), pathogen effectors can modulate host transcription either positively or negatively. (i) Activator-type effectors, such as transcription activator-like effectors (TALEs), bind host *cis*-regulatory elements (CREs) (e.g., EBEs) to induce disease-resistance (*R*) genes such as *Xa10*, triggering immune responses. (ii) Conversely, repressor-type effectors (e.g., MoHTR1/2) occupy *cis*-regulatory sites to suppress the expression of defense-related genes, resulting in effector-triggered susceptibility (ETS). **B** Post-transcriptional and translational control of plant immunity. **a**. Cap-independent translation: Purine-rich R-motifs and IRES-like elements in the 5′ untranslated regions (UTRs) of certain *R* genes allow translation initiation independent of the m^7^G cap, maintaining synthesis of R proteins when canonical translation is inhibited. **b**. Gene silencing: Small RNA pathways suppress immune transcripts through targeted degradation. DCL4 processes antisense transcripts of *SNC1* into 21-nt siRNAs, which are incorporated into the AGO1-RISC complex to further cut *SNC1* mRNA, thereby attenuating autoimmunity. **c.** Alternative splicing: Differential inclusion of exons or introns within *RPS4* pre-mRNA generates multiple transcripts that quantitatively modulate defense intensity. **d**. Alternative poly-adenylation (APA): Dynamic selection of proximal or distal poly(A) sites within *R* gene transcripts produces 3′ UTR isoforms, which may influence mRNA stability, localization, or translation. **e.** Upstream open reading frame (uORF)-mediated translational control: uORFs in the 5′-UTR (also known as 5′-leader) of *TBF1* repress its translation under resting conditions. Upon pathogen perception, this repression is relieved, enabling rapid TBF1 synthesis and activation of downstream defense programs. **C** Post-translational immune regulation. **a**. Phosphorylation: Activation of PRR complexes leads to BIK1 phosphorylation, which subsequently phosphorylates RBOHD and CNGC2/4, triggering reactive oxygen species (ROS) bursts and Ca^2^⁺ influx to propagate PTI signaling. **b**. Ubiquitination: In rice, pathogen-induced ubiquitination and degradation of ROD1 relieve its inhibition of OsTIR*,* thereby enhancing disease resistance. **c.** SUMOylation: Pathogen perception stimulates MC4-dependent cleavage of PROPEP1, releasing the peptide elicitor Pep1; MC4’s activity is fine-tuned by reversible SUMOylation/deSUMOylation, modulating DTI signaling. **d**. Proteolytic activation: The effector AvrPphB secreted by *P*. *syringae* cleaves the PBS1 kinase, which is subsequently recognized by the immune receptor RPS5, triggering a hypersensitive response. **e.** Nucleotide-binding leucine-rich repeat (LRR) receptor (NLR) activation: In ETI, Pik-1 and Pik-2 form a canonical NLR sensor-helper pair in rice. Pik-1, via its integrated heavy metal-associated (HMA) domain, directly recognizes the *Magnaporthe oryzae* effector Avr-Pik, thereby relieving Pik-2 from inhibition and activating downstream immune responses

Transcriptional regulation is an important step in plant immunity. A dynamic balance between transcriptional activation and repression prevents autoimmunity while precisely regulating the intensity and timing of immune responses (Fig. 1A). Nevertheless, the transcriptional circuitry underlying PTI remains only partially understood, limiting its utility in *R* gene engineering. In contrast, ETI involves highly specific transcriptional regulation of certain immune genes, a process in which pathogen effectors directly target promoters of host genes, leading to their transcriptional activation or repression. Notably, effector-targeted promoters often exhibit distinct epigenetic signatures—such as reduced DNA methylation or specific histone methylations. This specificity makes these immune proteins highly suitable for *R* gene engineering.

### Epigenetic regulation of plant defense responses

Epigenetic modifications play crucial roles in regulating plant defense. Compared with genetic mechanisms, epigenetic mechanisms offer reversible and context-dependent control of *R* gene expression, enabling plants to fine-tune the trade-off between growth and defense.

DNA methylation is among the most conserved epigenetic marks across eukaryotes and plays a critical role in transcriptional regulation. In plants, epigenetic regulation is particularly prevalent in *R* genes, where it can fine-tune defense responses. Whole-genome sequencing analyses in common bean have shown that nearly half of all *NLRs* are methylated. Notably, approximately 90 methylated *NLRs* are targeted by 24-nt small interfering RNAs (siRNAs) and exhibit reduced expression, consistent with RNA-directed DNA methylation (RdDM)-mediated transcriptional silencing (Richard et al. 2018; Wang et al. 2026). At the individual gene level, DNA methylation can directly modulate *R* gene expression. For example, *PigmS*, an allelic inhibitor encoded at the same locus, antagonizes PigmR signaling through heterodimerization. RdDM controls tissue-specific expression of *PigmS*: its promoter is hypermethylated and silenced in leaves, whereas hypomethylation in pollen permits expression. This epigenetic tuning enables tissue-specific activity of *PigmR*, thereby fine-tuning the trade-off between yield and immunity in rice (Deng et al. 2017). Similarly, the *R* gene *Xa21G* harbors 5mC modifications in its promoter that maintain transcriptional silence; artificial demethylation restores expression, which confers resistance to *Xoo* (Akimoto et al. 2007). In contrast, 5mC methylation can also positively regulate *R* genes. For instance, methylation of the *Pib* promoter enhances its inducible expression upon *Magnaporthe grisea* infection (Li et al. 2011). Beyond DNA methylation, additional epigenetic modifications, including chromatin remodeling and histone post-translational modifications (methylation, phosphorylation, ubiquitination, and ADP-ribosylation), may also contribute to fine-tuning immune responses (Hannan Parker et al. 2022; Yao et al. 2021; Zhang and Zeng 2020).

Given current technical challenges in achieving precise regulation of plant immune responses via epigenetic “switches”, we propose a potential strategy: a synthetic epigenetic on/off “switch” for targeted engineering of *R* genes (Zhang and Zhu, 2025). This approach involves fusing DNA demethylases (e.g., ROS1) with pathogen-inducible promoters (Gong et al. 2002). Upon *Xoo* infection, the system would relieve methylation-mediated silencing at *Xa21G* promoter, inducing its expression to enhance immune responses.

### Pathogen-induced transcriptional activation

During ETI, TALEs from pathogens recognize and bind to effector-binding elements (EBEs) in the promoters of executor *R* genes, thereby activating transcription and inducing cell death. Examples include TALEs that activate *Xa23, Xa7, Xa10,* and *Xa27* in rice, conferring resistance to bacterial blight and bacterial streak, as well as *Bs3* in pepper, which confers resistance to bacterial spot (Chen et al. 2021; Gu et al. 2005; Römer et al. 2010; Tian et al. 2014; Wang et al. 2014a, b). Additionally, the *R* gene *WeiTsing* (*WTS*) from *Brassica napus*, which confers resistance to clubroot, is rapidly induced in mesophyll cells upon infection by the protist pathogen *Plasmodiophora brassicae* (Wang et al. 2023). Thus, promoter structure and its cognate transcription factors (TFs) constitute vital components in the transcriptional regulation of plant immunity.

**Fig. 2:**
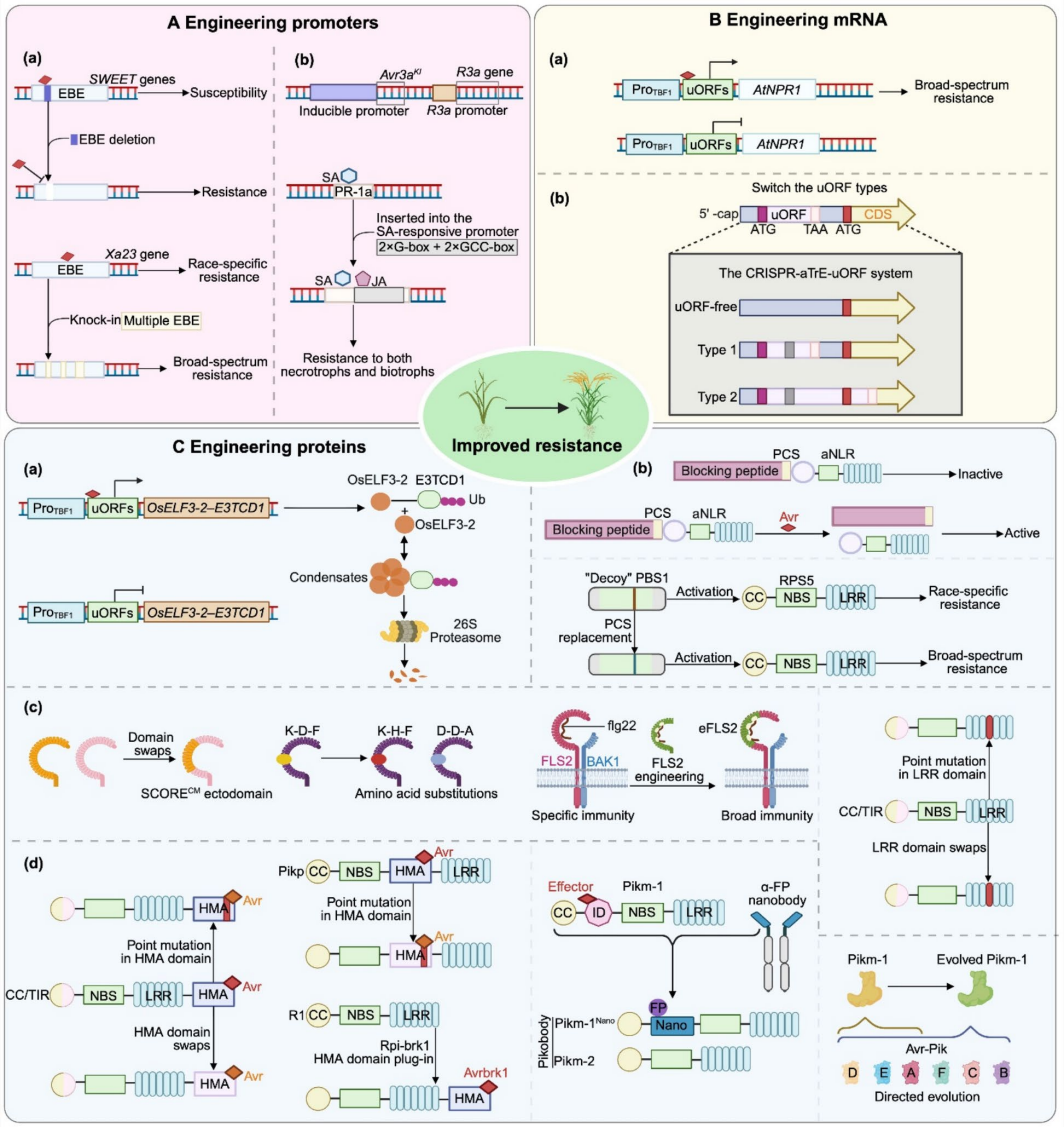
Strategies for engineering *R* genes in plants. **A** Engineering promoters. **a**. CRISPR-mediated *cis*-regulatory element engineering. Base editing of endogenous EBEs to abolish pathogen-inducible activation of susceptibility genes, as demonstrated for *SWEET* promoters in rice. Multi-EBE insertions (e.g., insertion of 10 EBEs in the *Xa23* promoter) broaden pathogen recognition. **b.** Synthetic promoter. 2 × S-4 × D-NpCABEcore cassette, to achieve late blight-specific expression in potato. Dual-hormone-responsive promoters are generated by inserting jasmonic acid (JA)-responsive motifs into the SA-responsive *PR-1a* promoter, enabling simultaneous defense against biotrophic and necrotrophic pathogens.. **B** Engineering mRNA. **a.** The heterologous expression of *AtNPR1* driven by the *TBF1* promoter in rice enhances resistance to rice bacterial blight and blast without compromising rice yield. **b.** uORF engineering. CRISPR-mediated editing of uORFs that naturally repress mORF translation fine-tunes R protein abundance, allowing balanced immune activation without growth penalties.. **(C)** Engineering proteins. **a.** Targeted protein-condensate degradation (TCD). A pathogen-responsive Pro_TBF1_-uORFs_TBF1_ cassette is used to provide dual transcriptional and translational control over a genetically encoded chimeric protein degrader (GE-CPD; Os-ELF3-2-E3TCD1) for targeting the endogenous susceptible protein OsELF3-2 for degradation through protein condensation. **b.** Protease cleavage-driven immunity-related protein engineering. The protease cleavage site (PCS) of the decoy PBS1, normally recognized by AvrPphB, was replaced with the PCS for the viral Nla-Pro protease. The engineered PBS1 conferred resistance to the corresponding virus. Additionally, protease-triggered activation re-engineers autoactive NLRs by separating functional domains with a pathogen protease-cleavable linker, confining immune signaling to the precise moment of protein cleavage, ensuring that the defense is activated only upon pathogen injection. **c.** LRR domain engineering. In PTI, LRR domain engineering of PRRs (e.g., FLS2 and SCORE) employs modular mutations or domain swaps to recognize new pathogens. During ETI, the introduction of mutations or domain swaps into the LRR domain of NLR proteins (e.g., Sr35 and Tm-2^2^) can confer broad-spectrum disease resistance in plants. **d.** Integrated domain (ID) engineering. HMA domain engineering in NLRs (e.g., RGA5, Pikp and R1) employs a modular strategy, enabling engineered receptors to recognize new pathogen effectors through structure-guided point mutations, domain swaps or domain insertions. Nanobody-fused NLRs are engineered by swapping native sensor domains (e.g., the HMA domain of Pikm-1) with nanobodies that recognize defined antigens (e.g., GFP). Co-expression with the cognate signaling partner (e.g., Pikm-2) reconstitutes the receptor complex and triggers downstream immune responses. Additionally, directed-evolution platforms (e.g., GRAPE) generate *Pikm-1* alleles with novel effector interfaces, while rational point mutations in the HMA domain expand the ligand-binding specificity of immune receptors

Transcriptional upregulation of target genes can be strategically engineered to function as immune “switches”. Some pathogen effectors, similar to TFs, bind to specific positive *cis*-regulatory elements (CREs) in the promoters of executor *R* genes, thereby activating the expression of* R* genes and triggering the plant immune response (Chen et al. 2021; Gu et al. 2005; Tian et al. 2014; Wang et al. 2014a, b; Wang et al. 2023). However, some CREs are located distantly from their target genes; this spatial separation can complicate precise promoter engineering. Recent advancements in genetic engineering have enabled the strategic design of synthetic pathogen-inducible promoters. These promoters are constructed by multimerizing minimal effector-responsive elements (12–24 bp in length) upstream of a core promoter, thereby achieving precise and inducible transcriptional control. For example, a) A combinatorial promoter module comprising 10 EBEs was designed to target ten distinct isolates of *Xoo* and *X. oryzae* pv. *oryzicola* (*Xoc*). Using genome-editing technologies, a 220-bp DNA cassette was inserted into the TATA-box region of the *Xa23* gene promoter, thereby conferring resistance to both bacterial blight and bacterial streak (Fig. 2A(a)) (Wang et al. 2024). The synthetic blight-inducible promoter 2 × S-4 × D-NpCABE, combined with the Avr3a allele has been engineered to generate transgenic potato lines that exhibit significantly enhanced resistance to *Phytophthora infestans* (Fig. 2A(b)) (Kauder et al. 2025). The natural SA-responsive *PR-1a* promoter has been engineered by inserting jasmonic acid (JA)-responsive elements (G-box and GCC-box), enabling it to respond to both SA and JA signaling pathways. This dual-responsive promoter allows a single gene to confer resistance to both necrotrophic and biotrophic pathogens (Fig. 2A(b)) (Li et al. 2023). The resulting germplasm offers a valuable genetic resource for broad-spectrum disease resistance.

Furthermore, the integration of CRISPR activation (CRISPRa) technology allows pathogen-inducible promoters to drive the expression of fusion proteins composed of dead Cas9 (dCas9) and transcription activators (e.g., VP64, VPR, or the SAM complex) (McLaughlin et al. 2025). These dCas9-based activator complexes have been demonstrated to specifically target the promoter regions of *R* genes, such as *SlPR-1* in tomato or *Pv-lectin* in common bean, thereby enhancing transcriptional activation and strengthening plant disease resistance (García-Murillo et al. 2023; Maximiano et al. 2025; McLaughlin et al. 2025).

### Pathogen-induced transcriptional repression

In the battle between plants and pathogens, pathogens have evolved effectors that bind to CREs in the promoter regions of target genes, thereby suppressing the expression of these genes and ultimately compromising plant immunity. Examples of such effectors include MoHTR1/MoHTR2 from *Magnaporthe. oryzae*, CRN108 and Avh23 from *Phytophthora sojae* (Kong et al. 2017; Lim et al. 2024; Song et al. 2015). Based on this, we can utilize pathogen‐delivered silencing effectors to suppress host susceptibility (*S*) genes and thereby confer disease resistance in plants. Using genome editing to introduce such CREs into the promoters of *S* genes can downregulate their expression upon pathogen infection, thereby enhancing plant disease resistance.

Moreover, we propose a novel strategy that integrates CRISPR interference (CRISPRi) technology, in which pathogen-inducible promoters drive the expression of proteins composed of dCas9 combined with single-guide RNAs (sgRNAs) (Khan et al. 2025). Currently, examples of CRISPRi-mediated enhancement of disease resistance remain limited. We propose targeting *S* genes for which partial suppression confers broad-spectrum resistance without yield penalty. For instance, partial downregulation of the rice *S* gene, *SWEET13*, via natural variation or induced mutations confers resistance to multiple pathogens (Oliva et al. 2019). Extending this strategy, we suggest employing the CRISPRi technology to enable pathogen-inducible, tunable repression of such *S* genes, thereby balancing resistance with yield. Alternatively, AI-driven direct promoter editing, combined with genome-editing technologies, has emerged as an effective strategy to downregulate *S* gene expression and confer broad-spectrum disease resistance in plants. By integrating AI-driven deep learning for promoter sequence analysis with multi-omics approaches, this method enables the precise deletion of CREs within the promoter, thereby fine-tuning gene expression and achieving a balanced growth-defense trade-off (Han et al. 2025a, b; Sha et al. 2023). This strategy is also applied to other plant genes, such as *SIMLO* in tomato (Zheng et al. 2016).

With recent advances in protein design algorithms, this method can be further enhanced by de novo design of DNA-binding proteins, which can now be computationally engineered to design proteins capable of recognizing target DNA with high affinity. We propose the rational design of DNA-binding proteins (promoter binders) that specifically interact with critical CREs within selected *S* or *R* gene promoters to either inhibit or activate their transcription (Glasscock et al. 2023). Upon pathogen infection, plants could a) induce expression of an inhibitory binder that reversibly suppresses *S* gene expression by competitively displacing endogenous activators, and b) activate a pathogen-inducible, designer DNA-binding protein that functions as a transcription factor to upregulate downstream plant defense-associated genes. This dual system strategy offers a flexible and tunable framework for engineering broad-spectrum disease resistance in plants through precise modulation of gene expression.

### Pathogen-inducible synthetic CREs

Two computational models have been reported for the de novo design of CREs. a) A deep learning model named PhytoExpr was trained on 600,000 genes from 17 plant species, coupled with over 6,000 matched transcriptomes. This model enables the design of novel regulatory DNA sequences with experimentally validated biological activity (Li et al. 2024a, b, c). b) Insulation strategies, deep learning models, and sequence design algorithms have been integrated to de novo design CREs that are absent from natural genomes. These synthetic sequences exhibit predictable transcriptional activity in both *Escherichia coli* and Chinese hamster ovary (CHO) cells (Wang et al. 2025a).

Similar models could be developed in plants, to enable precise regulation of *R* and *S* gene expression via the rational design of pathogen effector-responsive CREs. However, the function of every single base in non-coding DNA regions remains largely unresolved, and our understanding of how these regions quantitatively modulate gene expression is still limited.

Looking forward, the continued advancement of AI technologies combined with higher-quality omics datasets, may enable the design of highly specific CREs. Good targets could include vascular-specific CREs targeting diseases like bacterial wilt and Huanglongbing (HLB) (Hu et al. 2018; Wang et al. 2014a, b; Zhu et al. 2025a, b); epidermis-specific CREs that promote cuticle thickening or antimicrobial secretion to reinforce the plant’s first line of defense; and senescence-specific CREs activated during fruit ripening or leaf senescence to prevent postharvest diseases.

## Post-transcriptional level engineering

Post-transcriptional in this review regulation encompasses three principal mechanisms: (i) Regulation of mRNA stability and RNA interference (RNAi) or miRNA-mediated gene silencing, both modulating mRNA abundance and functional output (Fig. 1B(b)). (ii) Alternative splicing (AS) of pre-mRNA, which generates multiple mature mRNAs encoding distinct protein isoforms from a single gene (Fig. 1B(c)). (iii) Alternative polyadenylation (APA), where selection among alternative 3′-end poly(A) sites produces transcripts with variable 3′ untranslated regions (3’ UTRs) that differ in stability and translational efficiency (Fig. 1B(d)) (Wu et al. 2011). Based on these mechanisms, several potential strategies for engineering *R* genes can be proposed.

### Pathogen-induced RNA “switches”

Plant small RNAs (sRNAs) regulate immunity by post-transcriptionally silencing mRNAs via cleavage or translational repression. In Arabidopsis, siRNAs produced at the *RPP5* locus mediate post-transcriptional cosuppression of *SNC1* and neighboring *R* genes, preventing constitutive immune activation (Yi and Richards 2007), while in *Medicago truncatula*, miRNA-triggered phasiRNA production from *NLRs* generates secondary sRNAs that are loaded into AGO1 to cleave target transcripts (Zhai et al. 2011). In rice, miR168 targets AGO1, a key component of the RNA-induced silencing complex (RISC). Overexpression of miR168 suppresses AGO1, leading to increased disease susceptibility, delayed flowering, and reduced yield; conversely, expression of a target mimic for miR168 (MIM168) enhances resistance and improves yield (Wang et al. 2021). Meanwhile, miR1871 coordinates resistance and yield by regulating *OsMFAP1*. Inhibition of miR1871 increases *OsMFAP1* expression, promoting the number of productive tillers and enhancing PTI (Li et al. 2022). Additionally, miR160a confers broad-spectrum resistance to rice blast, bacterial blight, and sheath blight by suppressing multiple auxin response factor (*ARF*) genes, primarily *ARF8* (Feng et al. 2022). Together, these endogenous RNA silencing circuits help maintain immune homeostasis. Beyond intracellular regulation, sRNAs also mediate cross-kingdom defense. Plants transport specific sRNAs into pathogens such as *Botrytis cinerea* via exosome-like extracellular vesicles to silence pathogen virulence genes (Cai et al. 2018). This natural cross-kingdom RNA interference has been adapted into three delivery strategies for disease control: Spray-induced gene silencing (SIGS), in which exogenous dsRNA/siRNA is applied to foliage (Chen et al. 2025a, b); Host-induced gene silencing (HIGS), which expresses pathogen-targeted hairpin RNAs (hpRNAs) in planta (Nowara et al. 2010); and Microbe-induced gene silencing (MIGS), which uses rhizosphere microorganisms as delivery vectors (Wen et al. 2023).

Recent advances in RNA engineering have expanded both specificity and stability. The construction of intron-containing hpRNA (ihpRNA) vectors for plants can be efficiently facilitated by employing the OZ-LIC approach (Xu et al. 2010). Chimeric hpRNA structures incorporating conserved segments from four viral genes enable rice to resist four viruses simultaneously (Li et al. 2024a, b, c). Artificial microRNA (amiRNA) systems, meanwhile, utilize endogenous miRNA backbones (e.g., miR173) to guide cleavage of specific transcripts. For example, amiRNAs derived from the miR173 precursor have been engineered to target *RdRP*, *MP* and *CP* genes of tobacco mosaic virus and potato virus X (Gauthier et al. 2025). These tools could be extended to target *S* genes in the future: hpRNAs or amiRNA constructs directed against *OsSWEET14* in rice, *TaMlo* in wheat, or *DMR6* in tomato, when expressed under pathogen-inducible promoters, could potentially provide broad-spectrum resistance (Li et al. 2012; Thomazella et al. 2021; Wang et al. 2014a, b).

RNA stability motifs function as tunable post- transcriptional rheostats that complement sequence-specific silencing. N^6^-methyladenosine (m^6^A) modifications deposited by MTA-MTB writer complexes recruit ECT family YTH-domain readers, which in Arabidopsis have been implicated in regulating transcript stability and may promote decay of defense-related genes (Wei et al. 2018; Zhong et al. 2008). Consistent with this model, removal of m^6^A sites from defense-related gene transcripts can enhance their stability. Similarly, AU-rich elements (AREs) in 3’ UTRs recruit decay-promoting TZF proteins to fine-tune transcript abundance (Qu et al. 2014). Engineering these CREs could enable quantitative modulation of immune transcript levels, thereby optimizing the growth-defense trade-off.

### Pathogen-induced alternative splicing

Plants can enhance immune recognition and signal amplification by modulating AS of immunity-related genes. Pathogens are known to induce AS of plant *R* genes, thereby influencing disease resistance. In the model plant Arabidopsis, the *RPS4* gene undergoes AS upon recognition of the AvrRps4 effector from *Pseudomonas syringae*, conferring disease resistance (Zhang and Gassmann 2007). Similarly, AS of the potato *RB* gene produces a non-functional *RB_IR* isoform during normal growth, but switches to a functional *RB_CDS* transcript upon infection by *P. infestans*, enabling resistance (Sun et al. 2024). In tobacco, the *N* gene fine-tunes antiviral immunity by virus-triggered alternative inclusion of a 70-bp mini-exon, which alters isoform ratios and modulates the immune response (Dinesh-Kumar and Baker 2000). The *Pm4b* gene of wheat undergoes AS during powdery mildew infection to confer resistance (Sánchez-Martín et al. 2021). Conversely, the *OsNPR3* gene of rice is spliced during *Xoo* and *Xoc* infection, producing a truncated protein that increases susceptibility (Chen et al. 2025a, b; Sánchez-Martín et al. 2021). In addition, during *M. oryzae* invasion, oxidative stress promotes the production of the *OsMAPKKK18α* transcript without a retained intron, thereby enhancing immunity (Lu et al. 2025). AS in UTRs enables sophisticated post-transcriptional regulation of *R* genes. In the 5' UTR, AS alters the number and position of upstream open reading frames (uORFs), thereby modulating translation efficiency. For example, the barley *R* gene *Mla13* harbors two introns within the 5' UTR region of its pre-mRNA, generating five distinct transcript isoforms through AS. Notably, pathogen infection upregulates specific spliced variants associated with enhanced disease resistance (Halterman et al. 2003). Similarly, AS in the 3' UTR is also important in modulating immunity. The rice gene *OsNramp6* produces a long transcript isoform (*l-Nramp6*) that retains a 3' UTR lacking the miR7695 target site due to AS, thereby evading miR7695-mediated degradation during pathogen infection and sustaining the negative regulation of rice blast resistance by OsNramp6 (Campo et al. 2013).

Based on these mechanisms, two potential strategies for maintaining the balance between defense and growth are proposed. a) CRISPR-mediated knock-in of pathogen-inducible splicing elements can be used to engineer synthetic AS events that act as a trap for pathogens, generating highly active immune receptor isoforms upon infection. For instance, integrating effector-responsive intronic sequences or splicing enhancers into endogenous *R* genes could enable conditional expression of resistance isoforms, allowing the host to evade pathogen-mediated suppression of defense. b) A constitutively active splice variant can be designed that no longer depends on pathogen effectors or splicing factors for activation, yet still recognizes effectors and triggers immunity, as demonstrated for truncated *RPS4* isoforms (Zhang and Gassmann 2007). Modification of immune genes in these ways could prevent pathogens from suppressing host defense through the manipulation of AS. To avoid potential autoimmune responses, these variants should be expressed under tissue-specific or pathogen inducible promoters, to enable durable resistance without compromising growth.

### Pathogen-induced alternative polyadenylation

APA enables a single gene to produce transcripts with distinct 3' UTRs by utilizing different polyadenylation sites. This generates transcriptomic diversity and can influence mRNA stability, localization and translation efficiency. Genome-wide profiling in Arabidopsis reveals that about 70% of expressed genes undergo APA (Wu et al. 2011). APA has been observed in plants at different developmental stages and upon treatment with SA. Interestingly, 35% of the genes that undergo APA on SA treatment are related to biotic or abiotic stress responses, suggesting that APA may play a role in modulating the expression of immunity-related genes (Motion et al. 2015). In Arabidopsis, APA events have been observed in all 72 *R* genes, suggesting widespread transcript diversity, although the precise functional consequences remain to be elucidated (Tan et al. 2007). Insertion of the COPIA-R7 retrotransposon into the 5′ UTR of *RPP7* establishes H3K9me2 modification that regulates the APA of *RPP7*, fine-tuning transcript abundance of the immune receptor and consequently influencing disease resistance (Tsuchiya and Eulgem 2013). Similarly, CRISPR/Cas9-mediated editing of two APA sites within the human *CCND1* gene was performed, allowing analysis of the effects of distinct APA isoforms on *CCND1* expression and cell-cycle progression (Wang et al. 2018). These studies establish CRISPR-directed engineering of APA sites as a strategy for functional exploration of gene regulation.

To enhance plant immunity, genome-editing techniques can be exploited to insert transposon modules analogous to *COPIA-R7* into the 5′ UTR of other *R* genes. By modulating epigenetic modification levels, this approach can influence APA site usage in *R* genes, thereby promoting the generation of functional transcripts and enhancing disease resistance. Alternatively, targeted deletions, introduction of point mutations, or fragment replacement within proximal or distal APA sites in the 3′ UTR of *R* genes can be used to shift transcript isoforms toward longer or shorter variants, potentially increasing the abundance of immunity-related proteins. These strategies promote the expression of disease-resistant transcripts from *R* genes without altering the protein-coding sequences.

## Translational level engineering

Translational regulation of plant immunity modulates the abundance of defense proteins by controlling mRNA translation efficiency. Two mechanisms are involved: (i) cap-independent initiation, mediated by purine-rich RNA motifs (R-motif) in the 5′-leader of many *R* gene transcripts, which ensures sustained synthesis of defense proteins when cap-dependent translation is suppressed (Fig. 1B(a)) (Wang et al. 2022; Xu et al. 2017a, b; Yu et al. 2019; Zlotorynski 2022). (ii) uORFs repress translation of the downstream main open reading frame (mORF), thereby fine-tuning protein abundance (Fig. 1B(e)). These mechanisms prompt several potential strategies for engineering *R* genes.

### Pathogen-induced protein cap-independent translation

The R-motif is a purine-rich RNA element that drives cap-independent translation. In eukaryotes, the m^7^G cap at the 5’ end of an mRNA is essential for ribosome recruitment. During pathogen infection, decapping enzymes (DCPs) such as AtDCP2 remove the m^7^G cap, thereby promoting mRNA decay and reducing transcript levels (Yu et al. 2019). However, mRNAs that carry purine-rich R-motifs in their 5' UTRs can bypass this decapping and sustain cap-independent translation of immune regulators such as *TBF1* (Wang et al. 2022). Recently, the barley RNA-binding protein La1 was shown to specifically recognize the R-motif and promote translation of defense-related transcripts, enhancing antiviral immunity (Pi et al. 2025).

These findings can be translated into two engineering strategies for *R* genes. a) Introducing an R-motif immediately downstream of the native transcription start site, while leaving the endogenous promoter unaltered; upon pathogen infection, the R protein is cap-independently translated without altering steady-state mRNA levels. b) Combining an R-motif with a pathogen-inducible promoter creates a dual-layer regulatory system that restricts transcription to infection conditions and ensures cap-independent translation, thereby minimizing constitutive defense activation while enabling rapid R protein accumulation. With these approaches, it may be possible to achieve precise translational control that can be integrated with inducible promoters.

### Pathogen-induced protein translational derepression

uORFs are small open reading frames located within the 5' untranslated region (5' UTR) of mRNA (Mou et al. 2025; Xiang et al. 2023). Three classes of uORFs are currently recognized (Mou et al. 2025): first, suppressive uORFs, which attenuate mORF translation by impeding ribosome recruitment; second, translatable uORFs, whose short peptide products either stall ribosomes or function in trans to repress translation of distant mRNAs; and third, responsive uORFs, whose activity is selectively modulated by metabolites, RNA modifications, or trans-acting factors during development or stress. Among these, suppressive uORFs have been the most extensively studied. For instance, in Arabidopsis, the *TBF1* contains two uORFs within its 5′ leader sequence, which function as translational brakes: under normal conditions, these uORFs impede ribosomes and inhibit translation of the mORF. Upon pathogen infection, ribosomes bypass the uORFs, enabling rapid translation of *TBF1* and thereby enhancing immune responses (Xu et al. 2017a, b). Evidence suggests that HEM1 and CDC123, rather than GCN2, are involved in a uORF_TBF1_-mediated translational control pathway (Chen et al. 2023; Zhou et al. 2023).

Two strategies have been demonstrated for uORF engineering. a) Combining a pathogen-inducible promoter with a uORF to regulate translation. Upon pathogen infection, repression by the uORF is relieved, allowing rapid translation of the corresponding R protein and conferring disease resistance. For example, the transgenic cassette *Pro*_*TBF1*_*::uORF*_*TBF1*_*-AtNPR1* was shown to confer broad-spectrum disease resistance in rice (Fig. 2B(a)) (Xu et al. 2017a, b)*.* We propose that constructs such as *Ubi::uORF-Sr33* may enhance resistance to stem rust in wheat or *35S::uORF-CaRGA1* may improve pepper defense against the oomycete pathogen *Phytophthora capsici*. b) Precise modification of endogenous uORFs can fine-tune translation without introducing foreign DNA. Truncation of the uORF in *OsTBF1* by genome editing enables rice to maintain both stable yields while achieving broad-spectrum disease resistance (Tian et al. 2025). Similarly, editing the uORF of the rice heat shock factor gene *HsfA1* improves blast resistance under high temperatures (Qiu et al. 2025).

Furthermore, genome-editing techniques have been used to engineer uORFs through two complementary approaches. a) Achieving gradient-based downregulation of protein abundance by either creating de novo uORFs (insertion of ATG-uORF modules into the 5′ UTR) or extending endogenous uORFs via mutation of stop codons (Xue et al. 2023). b) Regulating the translation efficiency using the CRISPR-aTrE-uORF system, which constructs different uORF types (uORF-free, Type 1, Type 2) to fine-tune translation levels precisely (Fig. 2B(b)) (Tian et al. 2024). In summary, uORFs are promising regulatory elements for engineering plant immune “switches” offering precise, tunable and heritable control of disease resistance without compromising growth or yield.

## Post-translational level engineering

In plant immunity, post-translational modifications (PTMs) of proteins serve as key regulatory mechanisms that modulate immune responses, including phosphorylation, ubiquitination, SUMOylation, glycosylation, and acetylation. These PTMs can alter the conformation, stability, or subcellular localization of many immune receptors (e.g., PRRs or NLRs), thereby regulating their activity and facilitating the transmission of pathogen perception to downstream signaling. Additionally, protein cleavage, conformational changes and altered subcellular localization may further enhance signaling specificity. Collectively, these post-translational regulations form a multi-layered network that confers disease resistance across diverse environments in plants (Fig. 1C).

### Pathogen-induced activation of immune receptors

In plant immunity, plasma membrane-localized PRRs perceive PAMPs or DAMPs through their LRR or analogous domains, thereby initiating PTI. By contrast, NLRs detect effectors directly via C-terminal LRRs or “integrated domains” (IDs) such as heavy-metal-associated (HMA) domains. This recognition activates ETI through the N-terminal Toll/interleukin-1 receptor (TIR) or coiled-coil (CC) domain (Sun et al. 2020). The respective recognition surfaces dictate receptor–ligand specificity, and these interactions represent critical immune “switches”. Expanding the pathogen recognition spectrum by point mutations at key residues or by domain swapping provides a molecular framework for precise immune-receptor engineering in plants.

### LRR domain engineering

In PTI, PRRs rely on their LRR domains to detect PAMPs. However, pathogens frequently evade detection through epitope mutations. Recent advances have overcome this limitation by rational engineering of PRR ligand-binding domains. Mutagenesis of the FLS2 LRR domain has expanded its recognition spectrum (Li et al. 2025a, b; Zhang et al. 2025b). Likewise, targeted modification of the LRR scaffold in the selective cold shock protein receptor (SCORE) now enables recognition of cold-shock protein (CSP) variants secreted by diverse pathogens, including *Candidatus* Liberibacter asiaticus (citrus HLB), *Xanthomonas spp*., and root-knot nematodes (Ngou et al. 2025). Collectively, these results establish that direct engineering of PRR LRR domains can deliver broad-spectrum resistance. In ETI, structure-guided mutagenesis and domain swapping within the LRR domains of wheat immune receptors Sr33 and Sr50 have redirected their recognition specificity from *Puccinia graminis* f. sp. *tritici* effectors to new pathogen effectors, thereby conferring broad-spectrum disease resistance in wheat without triggering autoimmunity (Tamborski et al. 2023). Additionally, the endogenous LRR domains of TaSH1 and HvSH1 were swapped with that of Sr35, followed by structure-guided mutagenesis to engineer the effector-binding interface. These engineered receptors (e.g., TaSH1^Sr35^ and HvSH1^GOF^) are capable of recognizing AvrSr35 (Förderer et al. 2022). In tomato, high-throughput mutagenesis of the tomato CNL Tm-2^2^ LRR domain produced variants Tm-2^2−S723Y^ and Tm-2^2−N744D^, and each allele alone or the two combined confers robust resistance to both tomato brown rugose fruit virus and tobacco mosaic virus (Fig. 2C(c)) (Wang et al. 2025b).

### Integrated domain engineering

The HMA domain of plant NLR immune receptors is a heavy-metal-binding module that directly recognizes pathogen effectors and activates immunity. In rice, the C-terminal HMA domain of RGA5 directly recognizes the effectors Avr-Pia and Avr1-CO39, thereby relieving its inhibition of RGA4 and triggering immunity. Similarly, Pik-1 and Pik-2 constitute an NLR pair in which Pik-1 employs an HMA domain (inserted between the CC and NB-ARC domains) to recognize Avr-Pik. Allelic variation within the HMA domain of Pik-1, exemplified by Pikm allele, expands recognition to multiple Avr-Pik variants, thereby conferring broad-spectrum resistance. The HMA domain shows notable positional flexibility. In RGA5, the domain follows the LRR domain, whereas in Pik-1 and Pikm-1, it is located between the CC and NB-ARC domains. This positional flexibility has been exploited to engineer HMA domains that expand the recognition spectrum of NLRs (Cesari et al. 2013; De la Concepcion et al. 2021).

Structure-guided mutagenesis and domain swapping in the HMA domain of RGA5 have extended its binding specificity beyond Avr1-CO39 and Avr-Pia to include additional Avr effectors, thereby generating valuable disease-resistant germplasm (Zhang et al. 2024). Similarly, structure-guided mutagenesis in the HMA domain of Pikp yielded the Pikp^NK−KE^ mutant, which acquired recognition of additional effector variants, including Avr-PikA and Avr-PikE (De la Concepcion et al. 2019). In potato, the incorporation of the HMA domain from the NLR Rpi-brk1 into NLR R1 expended the resistance spectrum of R1 (Wang et al. 2025c). Furthermore, integrating high-throughput diversification of Pik-1 with protein language models has enabled recognition of previously undetectable Avr-PikC and Avr-PikF variants, while machine-learning-based screening facilitated rapid identification of new cognate* R* genes (Fig. 2C(d)) (Howard et al. 2025). Together, these strategies exemplify the power of structure-guided and AI-assisted design to expand disease resistance against both existing and emerging crop pathogens (Glasscock et al. 2023).

Collectively, these findings demonstrate that point mutations and domain remodeling can effectively expand the pathogen recognition spectrum of NLRs. Two innovative approaches further enhance this potential. a) Fusing plant-derived NLRs with nanobodies creates artificial immune receptors capable of recognizing specific pathogen effectors, thereby conferring targeted disease resistance (Kourelis et al. 2023). Moreover, AI-driven de novo antibody design has become feasible. Recent studies have employed the RFdiffusion structural diffusion model and large language models (LLMs) framework to de novo design antibodies that bind specific antigenic epitopes with atomic precision (Bennett et al. 2025; Wasdin et al. 2025). In the future, this approach is expected to enable the design of antibodies tailored to pathogen effectors. b) A novel directed evolution technique termed geminivirus replicon-assisted in planta directed evolution (GRAPE) mimics natural evolution to rapidly generate various NLR variants. For example, GRAPE successfully evolved the integrated domain of Pikm-1 to recognize six distinct Avr-Pik variants of the rice blast fungus (Fig. 2C(d)) (Zhu et al. 2025a, b). Moreover, advances in AI-driven de novo protein design are expected to enable the development of fully customizable immune “switches” capable of recognizing virtually any pathogen effector. Finally, these synthetic immune “switches” can be integrated into the crop genome via genome editing technologies, such as base editing or knock-in approaches.

### Pathogen-induced protein cleavage

Protein cleavage is a central regulatory mechanism in plant immunity that is exploited by both pathogens and plants. This includes non-specific protein cleavage, such as the secretion of the cysteine protease HopX1 by *P. syringae* pv. *tabaci*. HopX1 degrades JAZ proteins, thereby activating the JA signaling pathway, suppressing SA-dependent defense responses, and ultimately promoting infection (Geng et al. 2014). *P. syringae* pv. *tomato* secretes the metalloprotease AprA, which degrades flg22, thereby suppressing PTI (Pel et al. 2014). In contrast, specific protein cleavage also plays a regulatory role. For example, SUMOylation of PROPEP1 enhances its interaction with the protease MC4, thereby accelerating cleavage and release of Pep1, which amplifies danger-triggered immunity (DTI) (Zhang et al. 2025a; Zhou and Zhang 2020). Similarly, BAG3 maintains an autoinhibitory state via intramolecular domain interactions; upon viral infection, metacaspase MC4 cleaves BAG3 to release its N-terminal domain, which oligomerizes at the plasma membrane to form pores that restrict viral spread (Liang et al. 2025). MC4 also cleaves the RNA-binding protein La1, generating fragments that translocate to the cytoplasm and enhance R-motif mediated translation of defense-related mRNAs, thereby suppressing viral infection (Pi et al. 2025). Recent work has shown that the protease MC1 specifically cleaves Beclin 1 protein, thereby positively regulating autophagy initiation (Hu et al. 2025). Pathogens can exploit proteases to modulate immunity. Here, PsTry1, a trypsin-like serine protease secreted by the oomycete pathogen *P. sojae*, directly targets BAK1 in the plant apoplast. PsTry1 cleaves the extracellular domain of BAK1, thereby disrupting immune receptor complex assembly, blocking immune signal transduction, and inhibiting plant immune responses (Zhang et al. 2025c). Conversely, the effector AvrPphB secreted by *P. syringae* cleaves the PBS1 kinase, which is subsequently recognized by the immune receptor RPS5, triggering a hypersensitive response (HR) (Shao et al. 2003). These specific protease-mediated cleavage events provide valuable templates for *R* gene engineering.

Based on these mechanisms, scientists have developed a simple yet efficient strategy for the artificial design of plant *R* genes. Specifically, the protease cleavage site (PCS) of PBS1 recognized by AvrPphB was replaced with the PCS recognized by the viral Nla-Pro protease. The resulting modified PBS1 protein conferred resistance to the corresponding virus (Figure 2C(b)) (Kim et al. 2016). Similarly, substituting the native MC protease recognition site in proPeps with the PCS of Nla-Pro rendered transgenic Arabidopsis resistant to the corresponding virus (Fan et al. 2025). This protease cleavage-based strategy has been further extended to engineer NLRs. It involves expressing a fusion protein in plants comprising a polypeptide with a pathogen-derived PCS at its carboxyl (C) terminus fused to the N-terminus of an autoactive NLR (aNLR). In the absence of pathogens, the fused peptide suppresses aNLR activation, keeping the receptor in an inactive state. Upon pathogen invasion, the protease secreted by the pathogen specifically cleaves the fusion protein, releasing the aNLR and initiating immune signaling. This cleavage-dependent activation triggers a robust immune response, conferring resistance to multiple viruses in tobacco and soybean (Figure 2C(b)) (Wang et al. 2025d). Moreover, by clustering the cleavage sites recognized by multiple protease effectors from different pathogens, plants can be engineered to exhibit broad-spectrum disease resistance.

In summary, this strategy confers highly efficient, broad-spectrum, and stackable resistance to multiple viruses in tobacco, soybean, and Arabidopsis, providing a modular and universal “cleavage-release” immune “switch” for engineering disease resistance.

## Post-translational modifications

In plant immunity, PTMs serve as precise regulatory mechanisms modulating immune responses through distinct molecular modalities. Phosphorylation drives signal transduction downstream of PRRs and NLRs; ubiquitination directs selective proteolysis and turnover of immune regulators; and SUMOylation modulates nucleocytoplasmic shuttling of NLRs and transcription factors, fine-tuning defense responses and preventing autoimmunity. In parallel, glycosylation ensures proper folding and plasma membrane targeting of proteins such as PRRs while reinforcing extracellular defense proteins and cell wall integrity (Bethke et al. 2016; Jia et al. 2024). Acetylation coordinates immune responses both epigenetically through chromatin remodeling and metabolically via modulating enzymes involved in hormone biosynthesis (Kang et al. 2022; Mao et al. 2019). While glycosylation and acetylation establish foundational competence for immune signaling, phosphorylation, ubiquitination and SUMOylation constitute a more dynamic regulatory layer that directly regulates signal intensity and duration. Accordingly, we here focus on phosphorylation, ubiquitination and SUMOylation.

### Phosphorylation

Protein phosphorylation acts as a regulatory “switch” that orchestrates both PTI and ETI from perception to execution. In PTI, recognition of the bacterial flagellin peptide flg22 and the subsequent immune response rely on phosphorylation events (Li et al. 2014). Likewise, detection of fungal PAMP chitin relies on phosphorylation to trigger downstream immune signaling and defense response in rice (Yang et al. 2022). During ETI, the barley stripe mosaic virus TGB1 protein competitively occupies WAKL20 preventing phosphorylation of the NLR BSR1, which facilitates BSR1 resistosome formation and enhances antiviral immunity (Zhong et al. 2025). Conversely, pathogens secrete kinase-interfering effectors to disrupt these defenses. *Ralstonia solanacearum* RipAC binds the NLR co-chaperone SGT1 and inhibits its phosphorylation, thereby reducing NLR stability and compromising resistance (Yu et al. 2020). *P. syringae* HopAI1 directly dephosphorylates MPK3/6 and terminates the MAPK signaling cascade required for PTI (Zhang et al. 2007).

Protein phosphorylation during immune signaling is typically accompanied by conformational changes that alter protein structure and function, making the rational design of phosphorylation-dependent conformational “switches” challenging. Rather than engineering artificial “switches”, recent strategies have focused on leveraging naturally occurring phosphorylation-mediated regulatory mechanisms. Recent studies suggest that disease resistance can be enhanced by modulating phosphorylation-dependent regulation of NLRs. For example, phosphorylation of certain residues in NLRs or their interactors can promote activation (e.g., via SGT1-mediated stabilization), while dephosphorylation or prevention of phosphorylation may release auto-inhibition in some NLRs (e.g., BSR1) (Yu et al. 2020; Zhong et al. 2025). These approaches utilize genome editing to introduce amino acid substitutions that mimic or block phosphorylation, thereby modulating NLR conformational states and promoting immune response.

With the expanding repository of solved protein structures and the experimental validation of key phosphorylation sites (e.g., Ser938 in FLS2) through phosphoproteomics and related technologies, it is now increasingly feasible to use AI-derived modeling tools such as AlphaFold3 to predict phosphorylation-induced conformational changes (Abramson et al. 2024; Cui et al. 2024; Kadota et al. 2014). By integrating AI-predicted structures with kinetic modeling and treating phosphorylation rates as tunable parameters, future engineering pipelines could incorporate these phosphorylation sites into NLRs, facilitating the design of phosphorylation responsive conformational “switches” capable of detecting diverse pathogens and enhancing disease resistance.

### Ubiquitination

Protein degradation plays a fundamental role in maintaining the homeostasis of key regulatory factors across diverse biological processes, including plant immunity. Targeted protein degradation (TPD) is an emerging technique that directly edits the proteome, complementing traditional genome-editing tools such as CRISPR and RNAi (Niu et al. 2025). Analogous TPD technologies have revolutionized biomedical research. For instance, genetically engineered degradation tags enable reversible and tunable protein depletion, allowing rapid, tissue-specific knockdown of endogenous proteins in transgenic mice and thereby facilitating functional and disease-related studies (Nishimura et al. 2009). In cancer therapy, small-molecule degraders that simultaneously bind a target proteins and an E3 ubiquitin ligase promote selective ubiquitination and proteasomal degradation, effectively eliminating oncogenic drivers such as EGFR and suppressing tumor progression (Zhao et al. 2020). However, the application of comparable TPD strategies in plant systems remains limited.

Recently, the development of genetically encoded chimeric protein degraders (GE-CPDs) has marked a new advancement for targeted protein degradation in plants. For example, a self-degradable E3 ubiquitin ligase E3TCD1 was identified and engineered as an X-E3TCD1 fusion, which selectively degrades endogenous S proteins, enhancing crop disease resistance (Luo et al. 2025). In particular, their application to targeted protein-condensate degradation (TCD) enables the selective removal of proteins within specific condensate states, thereby advancing TPD technology and offering new possibilities to target R or S proteins with defined isoforms, post-translational modifications, subcellular localizations, or oligomeric states (Figure 2C(a)) (Niu et al. 2025).

Plants also exploit targeted degradation to relieve repression of immune responses. During rice blast infection, the E3 ubiquitin ligases RIP1 and APIP6 ubiquitinate and degrade the immune repressor ROD1, thereby releasing the NLR OsTIR and potentiating rice immunity (Wu et al. 2024). ROD1 can also act as a “degrader” with potential for synthetic manipulation. Building on these findings, two strategies are proposed. a) Pathogen-regulated stabilization of R protein. Fusion of the R protein to a “degrader” via pathogen-inducible cleavable peptides (PDC). Under normal conditions, the fusion is constitutively degraded. Upon pathogen infection, PDC cleavage releases the active R protein, triggering immunity. b) Pathogen-induced degradation of an R protein inhibitor by fusion of a degrader to an inhibitor protein. Upon pathogen perception, effector-induced ubiquitination degrades the inhibitor, thereby releasing and activating the R protein. This concept can be extended to pathogen-derived E3 ligases such as AvrPtoB from *P. syringae* (Janjusevic et al. 2006). Variants of AvrPtoB can ubiquitinate host NLR inhibitors, thereby enabling effector-triggered degradation of the repressor and consequent immune activation upon infection.

### SUMOylation

SUMOylation has emerged as a post-translational “switch” that spatially and quantitatively controls plant immunity. By reversibly modifying the activity, stability and subcellular localization of R proteins, SUMOylation fine-tunes the defense signaling and prevents autoimmunity. Perception of flg22 triggers FLS2-BAK1 complex formation and the release of BIK1 to initiate PTI; concurrently, FLS2 undergoes SUMOylation, thereby promoting receptor degradation and attenuating the duration of immune responses. This modification also imposes the signaling specificity on the co-receptor BAK1, while brassinosteroid signaling maintains BAK1 in a hypoSUMOylated state to favor growth, Pep1-dependent immune signaling promotes BAK1 SUMOylation, directing the kinase toward defense pathways (Orosa et al. 2018; Xia et al. 2025). Moreover, SA accumulation triggers NPR1 phosphorylation, which primes NPR1 for SUMOylation-dependent ubiquitination and subsequent degradation of monomeric NPR1 in the nucleus, thus relieving the suppression of SA-responsive defense genes to establish systemic acquired resistance (Zavaliev et al. 2020). Conversely, bacterial, oomycete and fungal effectors have evolved mechanisms to exploit the host SUMOylation system. *X. campestris* pv. *vesicatoria* secretes two type III effectors, AvrXv4 and XopD, that target the host SUMOylation system to suppress immunity. AvrXv4 encodes a YopJ-like cysteine protease with SUMO isopeptidase activity that cleaves SUMO-conjugates in planta, thereby reducing the accumulation of functional SUMOylated defense proteins. XopD, through its de-SUMOylase activity, specifically targets the transcription factor SIERF4 in tomato, thus suppressing ethylene-mediated immune responses and creating favorable conditions for pathogen growth (Kim et al. 2013; Roden et al. 2004). The HopG1 effector from *P. syringae* exploits the plant SUMOylation system to inhibit mitochondrial activity, thereby weakening immune defenses (Li et al. 2024a, b, c). Similarly, the effector protein MoHTR1 is SUMOylated by the host SUMOylation system, which maintains its stability and nuclear localization, promoting transcriptional reprogramming and suppressing rice immunity (Lim et al. 2024). Thus, SUMOylation operates at every stage of the plant-pathogen interaction, playing a pivotal role in fine-tuning plant immunity.

Despite its critical regulatory role, direct engineering of SUMOylation for disease resistance remains challenging. However, future approaches could leverage engineered SUMOylation of R proteins to modulate their activity, stability and subcellular localization, potentially enhancing disease resistance (Gou et al. 2017).

## Concluding remarks and perspectives

### Advantages of engineering plant immunity

Engineering of *R* genes represents an important advancement in plant immunity, leveraging synthetic biology to create "customized" disease resistance. This approach offers several key advantages: a) High precision and efficiency by facilitating direct integration of known *R* genes into elite cultivars, substantially shortening breeding cycles. b) Broad-spectrum resistance: Engineered *R* genes can be tailored to recognize a range of distinct pathogen effectors, providing broad-spectrum disease resistance. c) Cross species applicability: Using transgenic and genome-editing techniques, *R* genes from heterologous species can be stably integrated into crops, greatly expanding the repertoire of deployable resistance genes. Collectively, *R* gene engineering provides a powerful and efficient strategy for next-generation disease management in modern agriculture.

### Challenges and potential solutions of engineering plant immunity

Although the engineering of plant immunity has demonstrated significant advantages, its success still depends on the discovery of additional immune “switches”, which in turn relies on the continuous identification of novel *R* genes. The cloning of *R* genes and identification of immune “switches” have been greatly accelerated by the expanding availability of high-quality genomes, pan-genomes and mutant libraries. Furthermore, single-cell and spatial omics technologies are expected to facilitate high-resolution mapping of pathogen-induced or suppressed CREs in initially infected plant cells, which can be used as immune “switches” at the transcriptional level.

Engineering based on immune “switches” faces several major challenges: i) Components of immune “switches”, such as pathogen-induced promoters, carry the risk of triggering autoimmunity. ii) Temporal delays between the activation of immune “switches” and downstream immune responses can reduce efficacy. iii) The lack of tissue specificity in protein production can lead to tissue damage. These challenges partly stem from our incomplete understanding of the molecular mechanisms governing immune “switches”. To overcome these limitations, it is essential to dissect the mechanisms of immune “switches” at different levels. a) Transcriptional level: Single-cell and spatial transcriptomics allow gene expression to be analyzed at high resolution, thereby facilitating the identification of pathogen-responsive CREs. In Arabidopsis, these techniques enabled the discovery of PRIMER cells, offering the first insight into spatial and temporal dynamics of plant immune responses at the single-cell level, providing a novel framework for investigating and understanding plant immune “switches” (Nobori et al. 2025). Similar techniques in soybean, identified transcription factor-binding motifs enriched in specific cell types such as root-hairs, vascular-bundles and nodule cells. These resources can guide the discovery of tissue-specific transcriptional immune “switches” (Zhang et al., 2025). b) Post-transcriptional and translational level: Polysome and ribosome profiling (Ribo-seq) can define how uORFs and AS affect the expression of genes. c) Post-translational level: Cross-linking mass spectrometry and cryo-electron microscopy (cryo-EM) reveal conformational changes in R and Avr proteins before and after interactions, as demonstrated for ZAR1 and Sr35 (Förderer et al. 2022; Wang et al. 2019). However, high-resolution structures are still lacking for most plant proteins and pathogen effectors. Recent advances in AlphaFold-Multimer have enabled accurate prediction of effector–NLR interfaces, such as the RPP1–ATR1 and Sr35–AvrSr35 complexes, where models closely match cryo-EM structures (Förderer et al. 2022; Homma et al. 2023). To overcome challenges in determining structures of flexible or low-abundance plant proteins, high-throughput cryo-EM strategies can employ nanobody-fusion stabilization to enhance protein rigidity and reduce flexibility, thereby improving particle alignment and facilitating cryo-EM structure determination (Uchański et al. 2021; Yi et al. 2025). Together, these technologies deepen our mechanistic understanding of immune “switches”, and enable their rational design for optimized immune engineering.

**Fig. 3:**
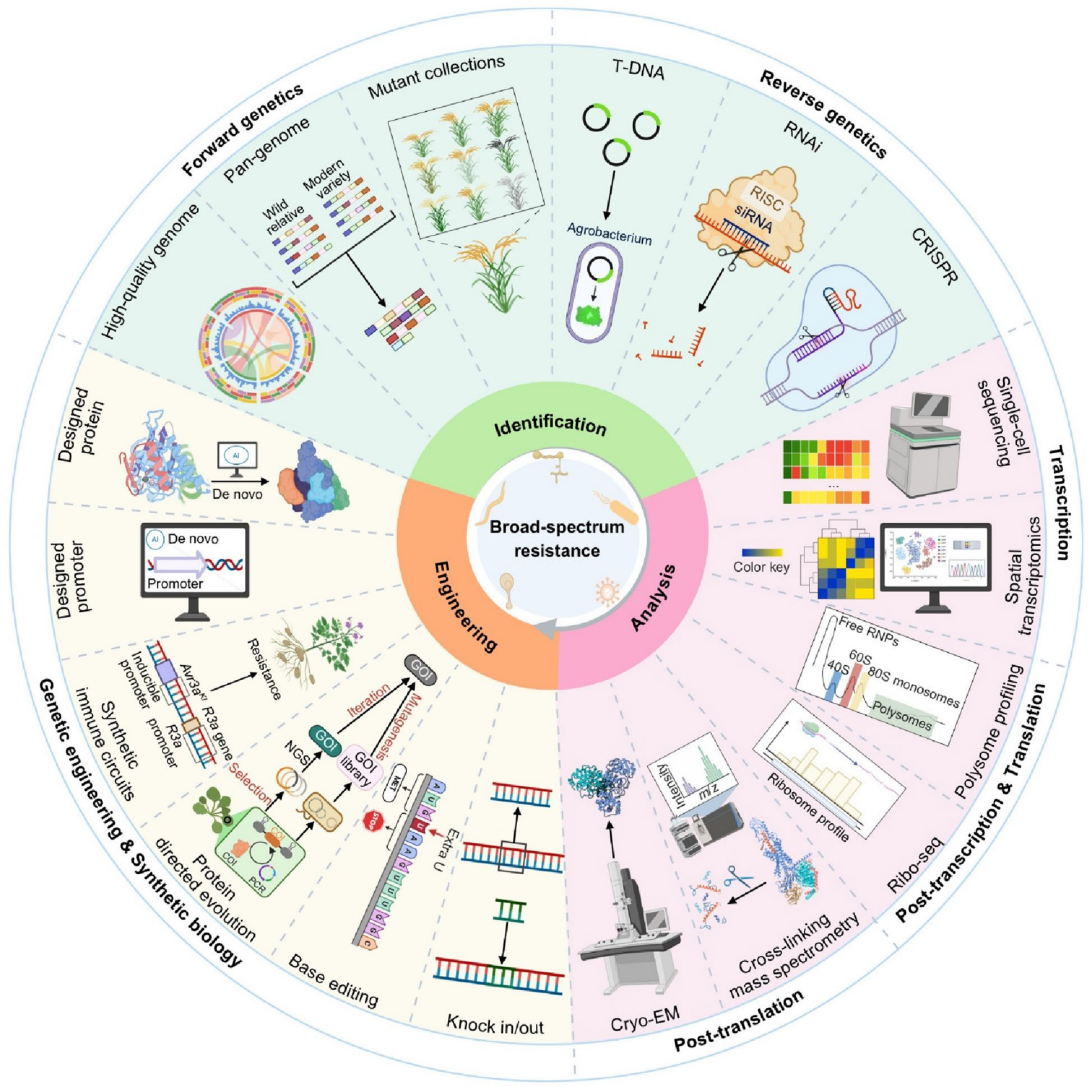
Technologies and tools enabling the discovery, analysis and engineering of plant *R* genes for optimized immune responses. **A** Identification of new *R* genes. Immune “switches” partly rely on the continuous discovery of more *R* genes. The continuous expansion of high-quality reference genomes and pan-genomic resources, together with saturated mutant libraries and advanced CRISPR/Cas or RNA-silencing platforms, accelerates the cloning and functional characterization of novel *R* genes across diverse plant species. **B** Multi-level analysis of immune “switch” mechanisms. Transcriptional level: Single-cell RNA-seq (scRNA-seq) and spatial transcriptomics provide high-resolution maps that resolve cell-type-specific expression patterns and identify CREs controlling *R* gene activity. Post-transcriptional and translational level: Polysome profiling and ribosome profiling (Ribo-seq) quantify translation dynamics and alternative splicing events that fine-tune *R* gene expression. Post-translational level: Cross-linking mass spectrometry and cryo-electron microscopy (cryo-EM) elucidate ligand-dependent conformational changes that activate or repress R protein complexes. **C** Engineering of *R* genes. Mechanistic insights derived from multi-level analysis are translated into precision engineering strategies. Genome-editing approaches using CRISPR/Cas-mediated knock-in/knock-out or base editing introduce targeted mutations that artificially modify endogenous immune “switches” enhancing plant immunity; synthetic biology frameworks reconstruct immune modules into modular components; directed evolution of these immune “switches” confers resistance against emerging pathogens or broadens the recognition spectrum of plant immune receptors; and AI-driven protein design enables de novo creation of synthetic immune “switches” not found in nature, allowing customized and programmable immune outputs

*R* gene engineering is increasingly empowered by rapid advances in modern biotechnology, enabling multiple innovative strategies. a) Genome editing: Techniques such as knockout, knock-in, and base editing are commonly used to introduce or fine-tune plant immune “switches”. Genome editing of promoter elements and regulatory sequences can reprogram plant immune responses. For example, modifying effector-binding elements to block pathogen effector binding, inserting new EBEs to broaden recognition of diverse strains, or altering upstream regulatory motifs to tune gene translation have all been shown to enhance or expand disease resistance in crops (Peng et al. 2017; Wang et al. 2024). b) Directed evolution: Directed evolution enables rapid expansion of the pathogen recognition spectrum by continuously optimizing immune “switches”. The GRAPE system allows high-throughput directed evolution in plants. The evolved NRC3 overcomes inhibition by the nematode effector SPRYSEC15, while the evolved Pikm-1 can simultaneously recognize six subtypes of Avr-Pik from the rice blast fungus, thereby broadening the spectrum of disease resistance (Zhu et al. 2025a, b). c) Protein design: Protein engineering enables the design or even de novo creation of immune “switches” to confer resistance to plant pathogens. For example, engineered “cleavage-activated” NLRs, carrying cleavage sites for six conserved viral proteases, confer resistance to over 100 types of viruses. Transferring domains between R proteins can broaden the spectrum of disease resistance, fusing nanobodies with plant NLRs produces synthetic immune receptors that confer resistance to specific plant pathogens. With the rapid advances in AI-driven protein design, de novo designed immune “switches” are likely to become increasingly common (Fig. 3) (Kourelis et al. 2023; Li et al. 2025a, b; Wang et al. 2025c, 2025d).

Pathogen engineering also represents an important consideration for exploiting immune “switches”. Continuous efforts to breed disease-resistant cultivars by targeting individual pathogen effectors are often short-lived, following a ‘boom-and-bust’ cycle. It is not uncommon for an *R* gene to lose effectiveness within a few years when new pathogen strains emerge and dominate the population. In East Africa, the stem rust pathogen race Ug99 rapidly overcame resistance conferred by the *Sr31* gene in many wheat varieties, leading to devastating outbreaks (Pretorius et al. 2000). Consequently, pathogen population genomic surveys are essential to maintain resistance and detect emerging virulence. Notably, a novel “endogenous sentinel” strategy has recently been proposed. This approach exploits interactions among microbes, host plants and pathogens to engineer plant endophytes that express effectors in response to generic pathogen-induced signals such as ROS. These effectors are then recognized by plant NLRs, triggering ETI in a generalized manner. By integrating multiple sentinel endophytes carrying conserved effectors and periodically replacing their alleles, this strategy activates plant immune “switches” from multiple angles. This strategy is expected to decelerate pathogen adaptive evolution, thereby extending the duration of resistance (Hou & Xu, 2025). Looking ahead, endophyte-engineered plant immunity (EEPI) achieves crop protection without modifying the host plant genome. This innovative approach offers a novel pathway for green and sustainable agriculture (Zhang et al. 2026).

In summary, *R* gene engineering is transitioning from time-consuming *R* gene cloning from limited genetic resources to a new phase of rational design of disease resistance, facilitated by immune “switches”. Leveraging forward and reverse genetic analyses, the increasing availability of high-quality plant (pan)genomes, multi-omics datasets and AI-driven design, de novo design immune “switches” can be identified to achieve precise regulation across levels from transcription to post-translational modifications. Concurrently, using transgenic and genome-editing strategies, crops such as rice and wheat have achieved efficient and durable disease resistance. Future efforts must establish pathogen population monitoring and early-warning systems, enabling real-time integration between “immune switches” and “pathogen population dynamics”. This approach would transform crop disease resistance from a passive to an active strategy, enabling precise disease control by engineering based on immune “switches”.

## Data Availability

Not applicable.

## Abbreviations

*R * genes: Disease-resistance genes
PAMPs: Pathogen-associated molecular patterns
SA: Salicylic acid
LRR: Leucine-rich repeat
TALE: Transcription activator-like effector
EBEs: Effector-binding elements
CREs: *Cis*-regulatory elements
CRISPRa: CRISPR activation
*S* genes: Susceptibility genes
sgRNAs: Single-guide RNAs
AS: Alternative splicing
UTRs: Untranslated regions
DCPs: Decapping enzymes
HMA: Heavy-metal-associated
CC: Coiled-coil
CSP: Cold-shock protein
GRAPE: Geminivirus replicon-assisted in planta directed evolution
HR: Hypersensitive response
aNLR: autoactive NLR
GE-CPDs: Gene-encoded chimeric protein degraders
ROS: Reactive oxygen species
siRNAs: small interfering RNAs
RISC: RNA-induced silencing complex
m^6^a: N^6^-methyladenosine
PTI: Pattern-triggered immunity
PRRs: Pattern-recognition receptors
ETI: Effector-triggered immunity
NLRs: Nucleotide-binding leucine-rich repeat receptors
AI: Artificial intelligence
TFs: Transcription factors
JA: Jasmonic acid
dCas9: Dead Cas9
CRISPRi: CRISPR interference
HLB: Huanglongbing
APA: Alternative polyadenylation
uORFs: Upstream open reading frames
IDs: Integrated domains
TIR: Toll/interleukin-1 receptor
SCORE: Selective cold shock protein receptor
LLMs: Large language models
DTI: Danger-triggered immunity
PCS: Protease cleavage site
TPD: Targeted protein degradation
ETS: Effector-triggered susceptibility
TCD: Targeted protein-condensate degradation
RdDM: RNA-directed DNA methylation
hpRNAs: hairpin RNAs
AREs: AU-rich elements
